# Mycoviruses in *Fusarium* Species: An Update

**DOI:** 10.3389/fcimb.2019.00257

**Published:** 2019-07-18

**Authors:** Pengfei Li, Pallab Bhattacharjee, Shuangchao Wang, Lihang Zhang, Irfan Ahmed, Lihua Guo

**Affiliations:** State Key Laboratory for Biology of Plant Diseases and Insect Pests, Institute of Plant Protection, Chinese Academy of Agricultural Sciences, Beijing, China

**Keywords:** *Fusarium*, mycovirus, mycovirus diversity, hypovirulence, mycovirus-host interactions, RNAi component

## Abstract

*Fusarium* is an important genus of plant pathogenic fungi, and is widely distributed in soil and associated with plants worldwide. The diversity of mycoviruses in *Fusarium* is increasing continuously due to the development and extensive use of state-of-the-art RNA deep sequencing techniques. To date, fully-sequenced mycoviruses have been reported in 13 *Fusarium* species: *Fusarium asiaticum, F. boothii, F. circinatum, F. coeruleum, F. globosum, F. graminearum, F. incarnatum, F. langsethiae, F. oxysporum, F. poae, F. pseudograminearum, F. solani*, and *F. virguliforme*. Most *Fusarium* mycoviruses establish latent infections, but some mycoviruses such as Fusarium graminearum virus 1 (FgV1), Fusarium graminearum virus-ch9 (FgV-ch9), Fusarium graminearum hypovirus 2 (FgHV2), and *Fusarium oxysporum* f. sp. dianthi mycovirus 1 (FodV1) cause hypovirulence. Rapid advances in various omics technologies used to elucidate genes or biological processes can facilitate an improved understanding of mycovirus-host interactions. The review aims to illuminate the recent advances in studies of mycoviruses in *Fusarium*, including those related to diversity, molecular mechanisms of virus-host interaction. We also discuss the induction and suppression of RNA silencing including the role of RNAi components as an antiviral defense response.

## Introduction

According to the tenth report of the International Committee for Taxonomy of Viruses (ICTV), fungal viruses or mycoviruses have been classified into seven linear dsRNA virus families and one linear dsRNA virus genus (*Chrysoviridae, Endornaviridae, Megabirnaviridae, Quadriviridae, Partitiviridae, Reoviridae, Totiviridae*, and *Botybirnavirus*, respectively), six linear positive-sense ssRNA virus families (*Alphaflexiviridae, Barnaviridae, Deltaflexiviridae, Gammaflexiviridae, Hypoviridae*, and *Narnaviridae*), one linear negative-sense ssRNA virus family (*Mymonaviridae*), two reverse transcribing linear ssRNA virus families (*Metaviridae* and *Pseudoviridae*), and one circular ssDNA virus family (*Genomoviridae*) (Lefkowitz et al., 2018). No dsDNA mycoviruses have been isolated from fungi.

Mycoviruses are generally transmitted vertically via spores and horizontally via anastomosis, with one exception, Sclerotinia gemycircularvirus 1 (formerly named Sclerotinia sclerotiorum hypovirulence-associated DNA virus 1), which can extracellularly infect fungi (Yu et al., 2013). Although many mycoviruses show latent infections, some cause phenotypic alterations that reduce or increase the virulence of the host (hypo- or hypervirulence) (Nuss, 2005; Pearson et al., 2010; Jiang et al., 2013; Ghabrial et al., 2015). Fungal viruses causing hypovirulence attract much attention as potential candidates for the biological control of phytopathogenic fungal diseases and provide an opportunity to understand the interactions between mycoviruses and plant-pathogenic fungal hosts (Nuss, 2005; Xie and Jiang, 2014; Ghabrial et al., 2015). Because most viruses lack an extracellular replication phase, researchers have developed many transformation and transfection techniques using cDNA infectious clones (Choi and Nuss, 1992; Chen et al., 1993; Zhang et al., 2016), dsRNA (Stanway and Buck, 1984; Kanhayuwa et al., 2015), *in vitro*-transcribed RNA transcripts (Chen et al., 1994; Chen and Nuss, 1999; Moleleki et al., 2003; Lin et al., 2007; Marzano et al., 2015), and purified virions (Hillman et al., 2004; Sasaki et al., 2007). These techniques have accelerated the identification of host and/or viral factor(s) involved in the interactions between mycoviruses and their hosts (Son et al., 2015). These methods can also be used to extend the experimental host ranges of some mycoviruses, without the restriction of fungal vegetative incompatibility (Son et al., 2015).

*Fusarium* is a widely dispersed filamentous phytopathogenic fungus genus of the phylum *Ascomycota*, which causes serious damage to many field, ornamental, forest, and horticultural crops (Sharma et al., 2018). Many *Fusarium* species also produce mycotoxins such as trichothecenes and fumonisins that cause health problems in humans and livestock, and some of the greatest economic impacts are associated with deoxynivalenol (DON) and its derivatives (Munkvold, 2017). Many species of *Fusarium* also act as hosts for mycoviruses. Although many mycoviruses have been reported from different species of *Fusarium*, only a few isolates have a hypovirulent effect on their host, which is one of the major challenges to using mycoviruses efficiently as a biocontrol agent against *Fusarium* species. This review discusses recent advances in research related to mycoviruses infecting *Fusarium* species.

## Diverse Viruses in *Fusarium*

Due to the advancement and widespread use of next generation sequencing (NGS) techniques, knowledge of the diversity of known mycoviruses has rapidly increased in the past few years. To date, complete genome sequences are available for 29 mycoviruses, which have been identified from different species of *Fusarium* (Table 1), including 17 dsRNA viruses from six established or provisionally designated families (*Crysoviridae, Megabirnaviridae, Partitiviridae, Totiviridae*, Alternaviridae, Fusagraviridae) and one unassigned dsRNA group, 11 (+)ssRNA viruses from five established or provisionally designated families (*Deltaflexiviridae, Hypoviridae, Narnaviridae*, Fusariviridae, and Tymoviridae) and one unassigned (+)ssRNA group, and one (–)ssRNA virus from the family *Mymonaviridae*.

**Table 1:** Fully-sequenced *Fusarium*-infecting mycoviruses.

**Genome type**	**Mycovirus**	**Established or proposed family**	**Host**	**RNA segment**	**Size of nucleotides**	**Accession no**.	**References**
dsRNA	FgV-ch9	*Chrysoviridae*	*F. graminearum* strain China 9	RNA1	3,581	HQ228213	Darissa et al., 2011, 2012
				RNA2	2,850	HQ228214	
				RNA3	2,830	HQ228215	
				RNA4	2,746	HQ228216	
				RNA5	2,423	HQ228217	
	FgV2	*Chrysoviridae*	*F. graminearum* strain 98-8-60	RNA1	3,580	HQ343295	Chu et al., 2004; Yu et al., 2011
				RNA2	3,000	HQ343296	
				RNA3	2,982	HQ343297	
				RNA4	2,748	HQ343298	
				RNA5	2,414	HQ343299	
	FodV1	*Chrysoviridae*	*F. oxysporum* f. sp. *dianthi* strain 116	RNA1	3,555	KP876629	Lemus-Minor et al., 2015
				RNA2	2,809	KP876630	
				RNA3	2,794	KP876631	
				RNA4	2,646	KP876632	
	FpgMBV1	*Megabirnaviridae*	*F. pseudograminearum* strain FC136-2A	RNA1	8,951	MH057692	Zhang et al., 2018
				RNA2	5,337	MH057693	
	FsV1	*Partitiviridae*	*F. solani* f. sp. robiniae strain SUF704	RNA1	1,645	NC_003885	Nogawa et al., 1996
				RNA2	1,445	NC_003886	
	FpV1	*Partitiviridae*	*F. poae* strain A-11	RNA1	2,185	NC_003883	Compel et al., 1999
				RNA2	2,203	NC_003884	
	FsPV2	*Partitiviridae*	*F. solani* f.sp. pisi	RNA1	1,950	LC006130	Osaki et al., 2015
	FaVV1	*Totiviridae*	*F. asiaticum* strain F16176	RNA1	5,281	MH615042	Li et al., 2019
	FgAV1	Alternaviridae	*F. graminearum* strain AH11	RNA1	3,524	MG254901	He et al., 2018
				RNA2	2,470	MG254902	
				RNA3	2,460	MG697236	
	FiAV1	Alternaviridae	*F. incarnatum* strain LY003-07	RNA1	3,548	MH899114	Zhang et al., 2019
				RNA2	2,514	MH899115	
				RNA3	2,498	MH899116	
	FpV2	Fusagraviridae	*F. poae* strain SX63	RNA1	9,518	KU728180	Wang et al., 2016a
	FpV3	Fusagraviridae	*F. poae* strain SX63	RNA1	9,419	KU728181	Wang et al., 2016a
	FvV1	Fusagraviridae	*F. virguliforme*	RNA1	9,402	JN671444	Marvelli et al., 2014
	FvV2	Fusagraviridae	*F. virguliforme*	RNA1	9,327	JN671443	Marvelli et al., 2014
	FgV3	Fusagraviridae	*F. graminearum* strain DK3	RNA1	9,098	NC_013469	Yu et al., 2009
	FgV4	Unassigned	*F. graminearum* strain DK3	RNA1	2,383	NC_013470	Yu et al., 2009
				RNA2	1,739	NC_013471	
	FgV5	Unassigned	*F. graminearum* strain HN1	RNA1	2,030	KX380787	Wang et al., 2017
				RNA2	1,740	KX380788	
(+)ssRNA	FgDFV1	*Deltaflexiviridae*	*F. graminearum* strain BJ59	RNA1	8,246	KX015962	Chen et al., 2016
	FgHV1	*Hypoviridae*	*F. graminearum* strain HN10	RNA1	13,023	KC330231	Wang et al., 2013
	FgHV2	*Hypoviridae*	*F. graminearum* strain JS16	RNA1	12,800	KP208178	Li et al., 2015
	FlHV1	*Hypoviridae*	*F. langsethiae* strain AH32	RNA1	12,839	KY120321	Li et al., 2017
	FcMV1	*Narnaviridae*	*F. circinatum* strain FcCa070	RNA1	2,419	KF803546	Martínez-Álvarez et al., 2014
	FcoMV1	*Narnaviridae*	*F. coeruleum* (MAFF No. 235976)	RNA1	2,423	LC006129	Osaki et al., 2015
	FgMV1	*Narnaviridae*	*F. globosum* (MAFF No. 237511)	RNA1	2,414	LC006128	Osaki et al., 2015
	FbMV1	*Narnaviridae*	*F. boothii* strain Ep-BL13	RNA1	2,802	LC425112	Mizutani et al., 2018
		*Narnaviridae*	*F. boothii* strain Ep-BL14	RNA1	2,801	LC425113	
		*Narnaviridae*	*F. boothii* strain Ep-N28	RNA1	2,802	LC425114	
	FgV1	Fusariviridae	*F. graminearum* strain DK21	RNA1	6,624	NC_006937	Chu et al., 2002; Kwon et al., 2007
	FgMTV1	Tymoviridae	*F. graminearum* strain SX64	RNA1	7,863	KT360947	Li et al., 2016
	FbLFV1	Unassigned	*F. boothii* strain Ep-BL13	RNA1	12,579	LC425115	Mizutani et al., 2018
(−)ssRNA	FgNSRV-1	*Mymonaviridae*	*F. graminearum* strain HN1	RNA1	9,072	MF276904	Wang et al., 2018

### Double-Stranded RNA Mycoviruses

#### Family *Crysoviridae*

Three members of the family *Crysoviridae* have been isolated from *Fusarium*, Fusarium graminearum virus-ch9 (FgV-ch9) from *F. graminearum* strain China 9 (Darissa et al., 2011, 2012), Fusarium graminearum virus 2 (FgV2) from *F. graminearum* strain 98-8-60 (Chu et al., 2004; Yu et al., 2011), and *Fusarium oxysporum* f. sp. dianthi mycovirus 1 (FodV1) from *F. oxysporum* f. sp. dianthi strain 116 (Lemus-Minor et al., 2015). The genome of FgV-ch9 consists of five dsRNAs, denoted as dsRNA1 to dsRNA5, 3,581, 2,850, 2,830, 2,746, and 2,423 bp in size, respectively, each containing a single open reading frame (ORF). These five dsRNAs are encapsidated separately in unequal amounts. Among those five dsRNAs, dsRNA1 encodes the viral RNA-dependent RNA polymerase (RdRp), 127 kDa in size, dsRNA2, and dsRNA3 encode two structural proteins, 94 and 93 kDa, resptectively. FgV-ch9 dsRNA4 and dsRNA5 encode polypeptides of unknown function, 91 and 79 kDa in size, respectively. The FgV2 genome also consists of five dsRNA segments referred to as dsRNA1 to dsRNA5, 3,580, 3,000, 2,982, 2,748, and 2,414 bp in size, respectively, each containing a single ORF flanked by conserved 5′ and 3′ untranslated regions (UTRs). FgV2 dsRNA1 encodes the viral RdRp, which is 127 kDa in size, while the other four dsRNAs encode proteins of unknown function (Yu et al., 2011). The FodV1 genome contains four dsRNA segments, 3,555, 2,809, 2,794, and 2,646 bp in length, respectively. Each of these segments contains a single ORF. FodV1 dsRNA1 and dsRNA3 encode an RdRp and a coat protein (CP), respectively, while dsRNA2 and dsRNA4 encode hypothetical proteins (named P2 and P4) with unknown functions (Lemus-Minor et al., 2015).

#### Family *Megabirnaviridae*

A member of the family *Megabirnaviridae* has been isolated from *Fusarium pseudograminearum* strain FC136-2A and is designated Fusarium pseudograminearum megabirnavirus 1 (FpgMBV1) (Zhang et al., 2018). The genome of FpgMBV1 consists of two dsRNA segments, L1-dsRNA (8,951 bp), and L2-dsRNA (5,337 bp), each containing two ORFs. L1-dsRNA encodes a CP of 131 kDa and an RdRp of 126 kDa, while L2-dsRNA encodes two proteins of 97 kDa and 31 kDa with unknown functions.

#### Family *Partitiviridae*

Three members of the family *Partitiviridae* have been isolated from *Fusarium*, Fusarium solani virus 1 (FsV1; synonym, FusoV) from *Fusarium solani* f. sp. *rohiniae* strain SUF704 (Nogawa et al., 1996), Fusarium poae virus 1 (FpV1; synonym, FuPO-1) from *Fusarium poae* strain A-11 (Compel et al., 1999), and Fusarium solani partitivirus 2 (FsPV2) from *Fusarium solani* f. sp*. pisi* (Osaki et al., 2015). The FsV1 genome consists of two dsRNAs, 1,645 and 1,445 bp in size, each containing one ORF. The large dsRNA encodes the viral RdRp, 60 kDa in size, while the small dsRNA encodes the CP, 44 kDa in size (Nogawa et al., 1996). The FpV1 genome consists of two dsRNAs, 2,185 and 2,203 bp in size. The two dsRNAs encode the viral RdRp and CP, 70 and 74 kDa in size, respectively (Compel et al., 1999). FsPV2 dsRNA1 (1,950 bp) in *F. solani* f. sp*. pisi* contains an ORF encoding an RdRp, 72 kDa in size. Sequence and phylogenetic analysis of the viral RdRps show that FsPV2 belongs to the genus *Alphapartitivirus*, family *Partitiviridae*. In general, partitiviruses possess two essential dsRNA genome segments: dsRNA1 (encoding the RdRp), and dsRNA2 (encoding the CP). However, the attempt to clone dsRNA2 of FsPV2 has failed (Osaki et al., 2015).

#### Family *Totiviridae*

A member of the genus *Victorivirus*, family *Totiviridae*, has been isolated from *Fusarium asiaticum* strain F16176 and is designated Fusarium asiaticum victorivirus 1 (FaVV1) (Li et al., 2019). The genome of FaVV1 consists of a single, linear dsRNA of 5,281 bp that contains two ORFs. ORF1 is predicted to encode a CP with a molecular mass of 79 kDa. ORF2 is predicted to encode an RdRp with a molecular mass of 92 kDa. Interestingly, the CP and RdRp of FaVV1 are most similar (77 and 75% identical, respectively) to those of Rosellinia necatrix victorivirus 1 (RnVV1), which infects the white root rot pathogen fungus *Rosellinia necatrix*. Both ORFs overlap with each other at a pentanucleotide “UAAUG,” and the overlapping pentanucleotide is one of the features of the genus *Victorivirus* (Li et al., 2019).

#### Family Alternaviridae

Two members of the newly proposed family Alternaviridae have been isolated from *Fusarium*, Fusarium graminearum alternavirus 1 (FgAV1) from *F. graminearum* strain AH11 (He et al., 2018), Fusarium incarnatum alternavirus 1 (FiAV1) from *F. incarnatum* strain LY003-07 (Zhang et al., 2019). The genome of FgAV1 possesses three dsRNA segments, dsRNA1 (3,524 bp), dsRNA2 (2,470 bp), and dsRNA3 (2,460 bp), each coding an ORF. FgAV1 dsRNA1 encodes an RdRp, 126 kDa in size, while dsRNA2 and dsRNA3 encode polypeptides of unknown function, 84 and 81 kDa in size, respectively. The RdRp of FgAV1 is most similar to Fusarium poae alternavirus 1 (FpAV1), with 98% (ORF1), 99% (ORF2), and 98% (ORF3) aa sequence identities (He et al., 2018), while the terminal sequences of FpAV1 have not been determined (Osaki et al., 2016). Compared to the genomic composition (4 dsRNA segments) of three other members of this family, namely, Alternaria alternata virus 1 (AaV1) (Aoki et al., 2009), Aspergillus mycovirus 341 (AMV) (Hammond et al., 2008), and Aspergillus foetidus mycovirus (AfMV) (Kozlakidis et al., 2013), FgAV1/AH11 and FpAV1 both lack the fourth segment. The genome of FiAV1 also consists of three dsRNA segments, dsRNA1 (3,548 bp), dsRNA2 (2,514 bp), and dsRNA3 (2,498 bp), each encoding a single ORF. FiAV1 dsRNA1 encodes an RdRp of 127 kDa, while dsRNA2 and dsRNA3 encode proteins with unknown functions (Zhang et al., 2019).

#### Family Fusagraviridae

Five members of the newly proposed family Fusagraviridae (Wang et al., 2016a) have been isolated from *Fusarium*, Fusarium poae dsRNA virus 2 (FpV2), and Fusarium poae dsRNA virus 3 (FpV3) isolated from the same *Fusarium poae* strain SX63 (Wang et al., 2016a), Fusarium virguliforme dsRNA mycovirus 1 (FvV1) and Fusarium virguliforme dsRNA mycovirus 2 (FvV2) isolated from *Fusarium virguliforme* (Marvelli et al., 2014), and Fusarium graminearum virus 3 (FgV3) isolated from *F. graminearum* strain DK3 (Yu et al., 2009). The respective genomes of FpV2 and FpV3 consist of a single dsRNA, 9,518 and 9,419 bp, respectively. Each contains two ORFs (ORF1 and ORF2), and encode a protein of unknown function and an RdRp, respectively (Wang et al., 2016a). A phytoreo_S7 domain is found downstream of the RdRp domain of the ORF2-coded proteins of both FpV2 and FpV3. The same shifty heptamer motif (GGAAAAC) is found immediately before the stop codon UAG of ORF1 in both FpV2 and FpV3, which could mediate programmed −1 ribosomal frameshifting (−1 PRF). The respective genomes of both FvV1 and FvV2 consist of a dsRNA, 9,402 and 9,327 bp, respectively, and contain two ORFs (ORF1 and ORF2) that overlap by 25 nt. The 25-nt overlap between ORFs 1 and 2 contain a slippery sequence (AAAAAAC) that is a−1 frameshift signal. ORF2 encodes an RdRp, while the function of ORF1 is unclear (Marvelli et al., 2014). The FgV3 genome also has a dsRNA (9,098 bp), and possesses two ORFs. ORF1 encodes a protein of unknown function, while ORF2 encodes an RdRp. A phytoreovirus S7 protein domain is also found downstream of the RdRp domain of ORF2 (Yu et al., 2009).

### Unassigned dsRNA Mycoviruses in *Fusarium*

Fusarium graminearum virus 4 (FgV4) has been isolated from *F. graminearum* strain DK3 (Yu et al., 2009). The FgV4 genome consists of two dsRNAs, 2,383 and 1,739 bp, respectively. FgV4 dsRNA1 contains a single ORF encoding the viral RdRp, while FgV4 dsRNA2 contains two putative ORFs coding for products of unknown function. Fusarium graminearum dsRNA virus 5 (FgV5) has been isolated from *Fusarium graminearum* strain HN1 (Wang et al., 2017). The FgV5 genome comprises two dsRNAs (dsRNA1 and dsRNA2), 2,030 and 1,740 bp, respectively. FgV5 dsRNA1 contains an ORF encoding an RdRp, 70 kDa. FgV5 dsRNA2 contains two ORFs (ORF2 and ORF3) that code for products of unknown function. Phylogenetic analysis indicates that FgV4 and FgV5 belong to a taxonomically unassigned dsRNA mycovirus group that is related to the families *Amalgaviridae* and *Partitiviridae* (Wang et al., 2017).

### Positive-Sense, Single-Stranded RNA Mycoviruses

#### Family *Deltaflexiviridae*

A member of the family *Deltaflexiviridae* has been isolated from *F. graminearum* strain BJ59 and is designated as Fusarium graminearum deltaflexivirus 1 (FgDFV1) (Chen et al., 2016). The FgDFV1 genome harbors a linear, positive-sense ssRNA, 8,246 nt in length excluding the poly (A) tails, and contains five putative ORFs. The largest ORF (ORF1) encodes a replication-associated polyprotein (RP) of 227 kDa, containing three conserved domains including viral RdRp, Hel, and Mtr. The four smaller ORFs (ORFs 2–5) encode four proteins of unknown function, 12, 13, 18, and 17 kDa, respectively.

#### Family *Hypoviridae*

Three members of the family *Hypoviridae* have been isolated from *Fusarium*, Fusarium graminearum hypovirus 1 (FgHV1) from *Fusarium graminearum* strain HN10 (Wang et al., 2013), FgHV2 from *Fusarium graminearum* strain JS16 (Li et al., 2015), and Fusarium langsethiae hypovirus 1 (FlHV1) from *Fusarium langsethiae* strain AH32 (Li et al., 2017). The FgHV1 genome consists of a linear, positive-sense ssRNA of 13,023 nt containing two ORFs (ORF A and ORF B). The 5′ proximal ORF A encodes a papain-like proteinase (p20), which is closely related to the Cryphonectria hypovirus 1 (CHV1)-encoded RNA silencing suppressor (RSS; p29) (Wang et al., 2016c). The 3′ proximal ORF B encodes a large polyprotein of 421 kDa with three conserved domains including papain-like protease (Pro), RdRp, and RNA helicase (Hel) (Wang et al., 2013). The FgHV2 genome consists of a linear, positive-sense ssRNA of 12,800 nt in size, containing a single ORF. The ORF encodes a large polyprotein of 446 kDa with three conserved domains, Pro, RdRp, and Hel, and a novel domain with homologous bacterial SMC (structural maintenance of chromosomes) chromosome segregation proteins (Li et al., 2015). The genome of FIHV1 contains a linear, positive-sense ssRNA, 12,839 nt in size, encoding a single ORF. The ORF encodes a polyprotein of 447 kDa, containing three conserved domains, Pro, RdRp, and Hel, which is a common feature of the family *Hypoviridae*.

#### Family *Narnaviridae*

Four members of the genus *Mitovirus* (family *Narnaviridae*) have been isolated from *Fusarium*, Fusarium circinatum mitovirus 1 (FcMV1) from *Fusarium circinatum* strain FcCa070 (Martínez-Álvarez et al., 2014), Fusarium coeruleum mitovirus 1 (FcoMV1) and Fusarium globosum mitovirus 1 (FgMV1) from *Fusarium coeruleum* (MAFF No. 235976) and *Fusarium globosum* (MAFF No. 237511), respectively (Osaki et al., 2015), and Fusarium boothii mitovirus 1 (FbMV1) from three different strains of *Fusarium boothii*: Ep-BL13, Ep-BL14, and Ep-N28 (Mizutani et al., 2018). The FcMV1 genome consists of a linear, positive-sense ssRNA of 2,419 nt, containing a single ORF that encodes an RdRp of 85 kDa. The genomes of FcoMV1 and FgMV1 consist of linear, positive-sense ssRNA, 2,423 and 2,414 nt, respectively, each containing a single ORF encoding an RdRp (86 and 84 kDa, respectively). FbMV1 genomes in strains Ep-BL13 and Ep-N28 are 2,802 nt in length and the genome in strain Ep-BL14 is 2,801 nt in length. These three virus strains share 98% nucleotide identity and each contains a single ORF encoding an RdRp.

#### Family Fusariviridae

A member of the newly proposed family, Fusariviridae has been isolated from *F. graminearum* strain DK21 (Chu et al., 2002; Zhang et al., 2014) and is designated Fusarium graminearum virus 1 strain DK21 (FgV1-DK21) (Yu et al., 2009). The genome of FgV1 consists of a linear, positive-sense ssRNA of 6,621 nt in length excluding the 3′-terminal poly (A) tail and contains four ORFs. The four ORFs (1-4) encode proteins with molecular masses of 174, 16.7, 6.2, 48.4 kDa, respectively, among which ORF1 encodes an RdRp, while the products of ORFs 2–4 reveal no obvious similarities to other viral sequences in the NCBI protein database (Kwon et al., 2007).

#### Family *Tymoviridae*

A member of the newly proposed genus Mycotymovirus, family *Tymoviridae* has been isolated from *F. graminearum* strain SX64 and is designated Fusarium graminearum mycotymovirus 1 (FgMTV1) (Li et al., 2016). The FgMTV1 genome consists of a linear, positive-sense ssRNA of 7,863 nt excluding the poly (A) tails, and contains four ORFs. ORF1 encodes a RP of 249 kDa, containing four conserved domains, viral RNA methyltransferase (Mtr), tymovirus endopeptidase (Pro), RdRp, and Hel. These four conserved domains are the common features of members of the family *Tymoviridae* (Martelli et al., 2002). ORFs 2–4 encode three hypothetical proteins of unknown function, 14, 13, and 19 kDa, respectively. FgMTV1 is the first tymo-like mycovirus isolated from a plant pathogenic fungus (Li et al., 2016).

### Unassigned (+)ssRNA Mycoviruses in *Fusarium*

Fusarium boothii large flexivirus 1 (FbLFV1) has been isolated from *Fusarium boothii* strain Ep-BL13 (Mizutani et al., 2018). The FbLFV1 genome consists of a linear, positive-sense ssRNA of 12,579 nt, and encompasses a single large ORF encoding a replicase of 444 kDa, containing three conserved domains, Mtr, Hel, and RdRp. There are also two regions of unknown function in the polypeptide with similarity to a PHA03247 domain found in herpesviruses (large dsDNA viruses). Sequence and phylogenetic analyses show that FbLFV1 belongs to a novel virus species that may form an independent genus, or even a novel family, in the order *Tymovirale*s (Mizutani et al., 2018).

### Negative-Sense, Single-Stranded RNA Mycoviruses

#### Family *Mymonaviridae*

A member of the family *Mymonaviridae* has been isolated from *Fusarium graminearum* strain HN1 and is designated Fusarium graminearum negative-stranded RNA virus 1 (FgNSRV-1) (Wang et al., 2018). The genome of FgNSRV-1 harbors a linear, negative-sense ssRNA of 9,072 nt, and contains five discontinuous but linear ORFs (ORF I-V), encoding five proteins (termed P I–P V) with molecular masses ranging from 6 to 221 kDa. Among the five FgNSRV-1 proteins, P II and P IV are related to the putative nucleoprotein N and large protein L of Sclerotinia sclerotiorum negative-stranded RNA virus 1 (SsNSRV-1) and SsNSRV-3 (Liu et al., 2014; Lee Marzano et al., 2016). The 3′ and 5′-ends of FgNSRV-1 RNA have perfect complementarity of the first six residues (3′-UCCUGC—GCAGGA-5′), which is a common feature among mononegaviruses (Chen et al., 2016).

## Effect of Viruses on *Fusarium*

Due to recent technological advances, a number of virus/fungal host systems can be established by curing viruses along with artificial introduction (Kondo et al., 2013; Suzuki, 2017). The vegetative growth, development, and physiological properties of host fungi are highly influenced by the harmful, beneficial, or neutral (little or no) effects of fungal viruses (Kondo et al., 2013). In this review, we mainly focus on the interplay between mycoviruses and *Fusarium* spp.

Mitigation of fungal virulence or “hypovirulence” is a good example of the harmful effect of several viruses on their host fungi. Therefore, fungal viruses could act as a potential candidate for the biological control of fungi (Hillman et al., 2018). The most studied example of reduced fungal virulence is CHV1/*C. parasitica* (Dawe and Nuss, 2013). In the case of *Fusarium* spp., four mycoviruses have harmful effects on fungal phenotypes. FgHV2, a novel hypovirus of the newly proposed genus Alphahypovirus, is associated with hypovirulence phenotypes, including reduced mycelial growth rate, conidia production, and DON concentration. In the virulence assay, the virus-free strain spreads more quickly from the inoculation sites to nearby spikelets than the virus-carrying strain JS16 (Li et al., 2015). FgV1, a member of the novel family Fusariviridae, has been reported to reduce the virulence of *F. graminearum*, delay mycelial growth, increase pigmentation, and reduce the production of mycotoxin (Chu et al., 2002). FgV-ch9, a member of the family *Crysoviridae*, is associated with hypovirulence of *F. graminearum* at high and medium viral dsRNA levels, including reduced mycelial growth rate and conidiation capacity, abnormal colony morphology, disorganized cytoplasm, and reduced virulence in wheat and maize plants (Darissa et al., 2012). FodV1, a member of the family *Chrysoviridae*, causes significant phenotypic alterations in the vegetative growth and virulence of the fungal host (Lemus-Minor et al., 2018).

In most cases, mycovirus infection causes little or no symptoms in the host (Ghabrial and Suzuki, 2009). For example, FgV3 and FgV4 infections do not cause any phenotypic change in the host *F. graminearum* (Lee et al., 2014). FgHV1 infection has mild or no effects on the fungal phenotypes of *F. graminearum* including morphology, mycelial growth, conidiation, and virulence or toxin production (Wang et al., 2013). FsV1 and FpV1 are also not associated with phenotypic changes in their hosts *F. solani* and *F. poa*e (Nogawa et al., 1996; Compel et al., 1999). Another two mycoviruses, FpV2 and FpV3, isolated from the same strain of *Fusarium poae*, do not show any deleterious effect on their host fungus (Wang et al., 2016a).

## Gene Regulations Associated With *Fusarium* Virus Infection

The proteome and transcriptome analysis of virus-infected fungi will help to elucidate the proteins and genes regulated by mycovirus infection that are involved in growth, development, and stress responses and direct further studies on the interactions between pathogenic fungi and viruses. The differential expression of *F. graminearum* proteins caused by FgV1 infection was investigated by two-dimensional electrophoresis with mass spectrometry (Kwon et al., 2009). Seven proteins, including sporulation-specific gene SPS2, triose phosphate isomerase, nucleoside diphosphate kinase, and woronin body major protein precursor, were induced or significantly upregulated by FgV1 infection. The significant decrease or down regulation of 16 proteins, including enolase, saccharopine dehydrogenase, flavohemoglobin, mannitol dehydrogenase, and malate dehydrogenase, caused by FgV1 infection was also identified. However, the number of proteins identified was insufficient to obtain a comprehensive understanding of the host response (Kwon et al., 2009; Cho et al., 2013). Therefore, genome-wide expression analyses were carried out using a 3′-tiling microarray in FgV1-infected *F. graminearum*. Genes associated with protein synthesis (such as ribosome assembly, nucleolus, and ribosomal RNA processing) and genes required for transcription and signal transduction (such as fungal-specific transcription factors and cAMP signaling), were significantly up-regulated in fungal host cells, which seems to be related to virus replication. In contrast, significant down-regulation of genes required for metabolism (such as carboxylic acids, aromatic amino acids, nitrogen compounds, and polyamines) and transporting systems in a fungal host containing the virus appears to be related to the host defense mechanism and fungal virulence (Cho et al., 2012, 2013). Furthermore, a comprehensive genome-wide gene expression analysis elucidated completely distinct expression patterns of *F. graminearum* transcriptomes in response to infections by two hypovirulent mycoviruses (FgV1 and FgV2) and two non hypovirulent mycoviruses (FgV3 and FgV4), respectively. Interestingly, changes in the host transcriptome caused by different mycoviruses are not always correlated with observed host phenotypes. The fungal host transcriptome was more affected by FgV1 and FgV4 infections than by FgV2 and FgV3 infections. In addition, 12 differentially expressed genes were identified in response to all four mycovirus infections, but functions of most of these genes are still unknown (Lee et al., 2014). In the FgHV1-infected *F. graminearum* strain, enriched genes related to redox regulation are differentially expressed. It has been demonstrated that FgHV1-encoded p20 could induce H_2_O_2_ accumulation and a hypersensitive response in *Nicotiana benthamiana* leaves (Wang et al., 2016c).

Subsequently, the hexagonal peroxisome protein FgHex1 was screened using transcriptional and proteomic analyses. By generating *FgHEX1* gene deletion and overexpression mutants, it was indicated that the *FgHEX1* gene plays a direct role in the asexual reproduction and virulence of *F. graminearum* and facilitates viral RNA accumulation in the FgV1-infected host fungus (Son et al., 2013). To investigate the molecular mechanism underlying the production of *FgHEX1* and the replication of FgV1 viral RNA, electrophoretic mobility shift assays (EMSA) was conducted with recombinant FgHex1 protein and RNA sequences derived from various regions of FgV1 genomic RNA. These analyses demonstrated that the FgHex1 functions in the synthesis of both strands of FgV1 RNA and therefore in FgV1 replication probably by specifically binding the FgV1 genomic RNA. This is the first report about the regulation of viral RNA replication by a fungal cellular protein that can directly bind to viral genomic RNA (Son et al., 2016). Otherwise, another host gene, the putative 3′(2′),5′-bisphosphate nucleotidase gene, *FgHal2*, in *F. graminearum* is down-regulated following FgV1 infection. The possible function(s) of *FgHal2* was investigated in *F. graminearum* using gene deletion and gene over-expression mutants. It was found that deletion of *FgHal2* reduced conidiation, mycelial growth, and the production of secondary metabolites. Moreover, deletion of *FgHal2* decreased viral RNA accumulation and the vertical transmission of FgV1 via conidia. Together, these results indicate that *F. graminearum* can down-regulate one of its major multifunctional genes, *FgHal2*, in response to FgV1 infection (Yu et al., 2015). Recently, an mRNA binding protein (named virus response 1, *vr1*) was identified to be involved in symptom expression in *F. graminearum*. Downregulation of *vr1* in the virus FgV-ch9 infected fungus and *vr1* deletion evoke virus-infection like symptoms while constitutive expression overrules the cytopathic effects of the virus infection. Intriguingly, the presence of a specific viral structural protein P3 is sufficient to trigger the fungal response, i.e., *vr1* downregulation, and symptom development similar to virus infection. Hence, *vr1* represents a fundamental host factor for the expression of virus-related symptoms and helps to understand the underlying mechanism of hypovirulence. The advancements in understanding fungal infection and response may aid biological pest control approaches using mycoviruses or viral proteins to prevent future *Fusarium* epidemics (Bormann et al., 2018).

## Rnai in *Fusarium* Responding to Viral Infection

RNA silencing or RNA interference (RNAi) functions as an antiviral defense mechanism in eukaryotic organisms. The core elements of the cross-kingdom RNA silencing defense response consists of conserved ribonucleases: members of the Dicer-like (DCL) and Argonaute-like (AGO) protein families. Dicer nucleases recognize viral double-stranded and structured RNAs and use the associated RNase III-type activity to process these RNAs into small RNAs of 21–24 nts in length, termed virus-derived small (vs.) RNAs. The vsRNAs are then incorporated into an effector complex termed the RNA-induced silencing complex (RISC) with the aid of an Argonaute family protein. One strand of the vsRNA is degraded and the remaining guide strand targets the effector complex to the cognate viral RNA, which is then cleaved by the Argonaute-associated RNAse H-like activity. Subsequently, the antiviral RNA silencing response is further amplified by host RNA-dependent RNA polymerases (RdRPs) (Ding, 2010; Nuss, 2011). To combat RNA silencing-based antiviral defense, viruses of plants, insects, mammals, and fungi encode proteins, designated viral suppressors of RNA silencing (VSR), that employ a variety of mechanisms to suppress RNA silencing pathways. RNA silencing and viruses against host RNA silencing have been well-characterized in the *Cryphonectria parasitica*-hypovirus system (Ding, 2010; Nuss, 2011). More encouragingly, the roles of core components of the RNAi pathway have been also been gradually revealed in *F. graminearum*.

*F. graminearum* contains genes encoding two dicer proteins (FgDicer1 and FgDicer2), two Argonaute proteins (FgAgo1 and FgAgo2), and five RdRp proteins (FgRdRp1 to 5). Chen et al. showed that RNAi machinery was not involved in growth, abiotic stress and pathogenesis in *F. graminearum*. In addition, it was demonstrated that the hairpin RNA (hpRNA) can efficiently silence the expression level of target gene and the argonaute protein FgAgo1 and dicer protein FgDicer2 played a critical role in the hpRNA mediated gene silencing process (Chen et al., 2015). FgDicer2 was also involved in small RNA (sRNA, 17–40 nt) transcription and micro-like-RNA (milRNA) generation in this fungus (Chen et al., 2015). Strikingly, a recent study showed that FgDicer2 is the primary DCL component for defense against viroids in *F. graminearum* (Wei et al., 2019). Furthermore, a combined analysis of functional genetics and deep sequencing of small non-coding RNA (sRNA), mRNA, and the degradome indicate that the sex-specifically induced exonic small interference RNA (exsiRNA)-mediated RNA interference (RNAi) mechanism has an important role in fine-tuning the transcriptome during ascospore formation in *F. graminearum*. It is FgDicer1 and FgAgo2 that primarily mediate the sex-specific RNAi pathway. Each fungal species appears to have evolved RNAi-based gene regulation for specific developmental stages or stress responses. This study provides new insights into the regulatory role of sRNAs in fungi and other lower eukaryotes (Son et al., 2017).

To preliminarily elucidate the RNA silencing mechanism of the *F. graminearum*/hypovirus system, the properties of sRNAs in FgHV1 and FgHV2 were analyzed by using HiSeq deep sequencing in *F. graminearum*. The length distributions of *F. graminearum* sRNAs were altered by hypoviral infection. Potential milRNA candidates were differentially expressed between the hypovirus-free and hypovirus-infected library types. The 1,831,081 and 3,254,758 total reads generated from the FgHV1 and FgHV2 genomes in *F. graminearum* yielded the first high-resolution sRNA maps of fungal viruses. In particular, FgDicer1 and FgRdRp5 were predicted targets of FgHV1- and FgHV2-derived siRNAs, possibly revealing a novel anti-RNA silencing strategy employed by mycoviruses (Wang et al., 2016b). To investigate the contributions of RNAi components to the antiviral response against Fusarium graminearum viruses (FgV1, 2, and 3), reverse genetics and virus-derived small RNA profiling were used. FgV1–3 infection differentially induces the gene expression of RNAi components in *F. graminearum*. *FgDICER2* and *FgAGO1* transcripts accumulated at lower levels following FgV1 infection than following FgV2 or FgV3 infection. *FgAGO1* can efficiently robust RNAi response against FgV1 infection, but the functions of two dicer genes, i.e., *FgDICER1* and *FgDICER2*, might be partially redundant in response to FgV 1–3 infections. These results show that *F. graminearum* developed more complex and robust RNA silencing system against mycoviruses (Yu et al., 2018).

## Conclusion and Prospects

The characterization of the viruses isolated from *Fusarium* mentioned above has enhanced our understanding of the great diversity of mycoviruses. Most *Fusarium* mycoviruses have double-stranded or single-stranded, positive- or negative-sense RNA genomes. No DNA viruses have been isolated from *Fusarium* species. Additionally, purified virions of *Fusarium* mycoviruses can be obtained in large quantities and of high quality and can serve as good material for structural analysis, which will promote a better understanding of mycovirus assembly, function, evolution, and its uses in nanotechnological applications (Ghabrial et al., 2015; De Ruiter et al., 2019).

Fungal viruses causing hypovirulence can be used as potential biocontrol agents of phytopathogenic fungal diseases, which are always restricted by fungal vegetative incompatibility (Son et al., 2015). Recent studies have demonstrated that some mycoviruses can be transmitted between vegetatively incompatible strains, particularly SsHADV-1, Sclerotinia sclerotiorum partitivirus 1 (SsPV1), Sclerotinia sclerotiorum mycoreovirus 4 (SsMYRV4), and Sclerotinia sclerotiorum deltaflexivirus 2 (SsDFV2) (Yu et al., 2013; Xiao et al., 2014; Wu et al., 2017; Hamid et al., 2018). In addition, these mycoviruses can cause hypovirulence. Their hypovirulent properties suggest that SsHADV-1, SsPV1, SsMYRV4, and SsDFV2, have potential for controlling plant disease in the field. Therefore, further research is required to clarify whether the transmission of *Fusarium* mycoviruses can occur and their corresponding transmission efficiency between vegetative-incompatible individuals or interspecific fungi.

*Fusarium graminearum* has emerged as a good model system for studying mycovirus–host interactions, similar to three other host-virus interaction systems: *C. parasitica*–mycovirus, *S. sclerotiorum*–mycovirus, and *R. necatrix*–mycovirus. There are some advantages of using *F. graminearum*–mycovirus for such studies. For example, the genome of the *F. graminearum* strain PH-1 is sequenced and is available publicly, allowing RNA-seq-based, genome-wide expression analysis and modification of targeted gene disruptions. Additionally, protoplasts of *F. graminearum* are easy to prepare and store and are competent in genetics transformation systems with DNA. Infectious cDNA clones have been successfully used in a few fungal species, including *C. parasitica, Diaporthe ambigua*, and *Sclerotinia sclerotiorum* (Choi and Nuss, 1992; Chen et al., 1993, 1994; Chen and Nuss, 1999; Moleleki et al., 2003; Lin et al., 2007; Marzano et al., 2015; Zhang et al., 2016), but not so far in *F. graminearum*. The construction of infectious cDNA clones can be used to explore the properties of mycoviruses and employ mycoviruses as vectors to introduce genes deleterious to the fungal host (Pearson and Bailey, 2013).

Future studies should focus on systematically identifying viral and host factors important for the interactions between mycoviruses and their hosts, especially crucial determinants responsible for the phenotypic changes (including hypovirulence) and reduction of *Fusarium* mycotoxin production caused by mycovirus infection.

## Author Contributions

SW, LZ, and IA performed the literature search and prepared the manuscript table. LG drafted and revised the manuscript. PL and PB contributed equally to writing the manuscript. All authors revised and agree with the final manuscript version.

### Conflict of Interest Statement

The authors declare that the research was conducted in the absence of any commercial or financial relationships that could be construed as a potential conflict of interest.
